# Birth Weight Is Associated With Kidney Size in Middle-Aged Women

**DOI:** 10.1016/j.ekir.2021.08.029

**Published:** 2021-09-07

**Authors:** Bjørn Steinar Lillås, Tor Hatlestad Qvale, Blazej Konrad Richter, Bjørn Egil Vikse

**Affiliations:** 1 Department of Medicine, Haugesund Hospital, Haugesund, Norway; 2 Department of Clinical Medicine, University of Bergen, Bergen, Norway; 3 Department of Radiology, Haugesund Hospital, Haugesund, Norway; 4 Department of Radiology, Stavanger University Hospital, Stavanger, Norway

**Keywords:** kidney size, low birth weight, magnetic resonance imaging, ultrasonography

## Abstract

**Introduction:**

Low birth weight (LBW) is associated with increased risk of kidney disease due to lower nephron endowment leading to hyperfiltration and subsequent nephron loss. Kidney size is commonly used as a proxy for nephron number. We compared kidney volume measured by magnetic resonance imaging (MRI) with measured glomerular filtration rate (mGFR) in adults with either normal birth weight (NBW) or low birth weight (LBW).

**Methods:**

Healthy individuals aged 42 to 52 years with LBW (1100−2300 g) and NBW (3500 −4000 g) were invited to participate. The GFR was measured using plasma clearance of iohexol. Kidney volume was measured on magnetic resonance images using axial T2 images and coronal T1 images with fat saturation without contrast enhancement; calculations were performed according to the ellipsoid formula π/6 × length × width × depth.

**Results:**

We included 102 individuals (54 LBW and 48 NBW). Total kidney volume was 302 ± 51 ml for female NBW vs 258 ± 48 ml for female LBW individuals (*P* = 0.002). For male individuals, total kidney volume was 347 ± 51 ml vs. 340 ± 65 ml (*P* = 0.7). The mGFR was significantly associated with kidney volume, with *r* = 0.52 (*P* < 0.001) for women and *r* = 0.39 (*P* = 0.007) for men. A mediation analysis showed that the association between birth weight and mGFR (significant in total sample and women) was mediated by kidney volume.

**Conclusion:**

Healthy female individuals born with LBW have smaller kidneys than healthy females born with NBW. The previously shown associations between LBW and lower mGFR in adult women might be explained by smaller kidney volume.

## Graphical abstract

**Figure:**
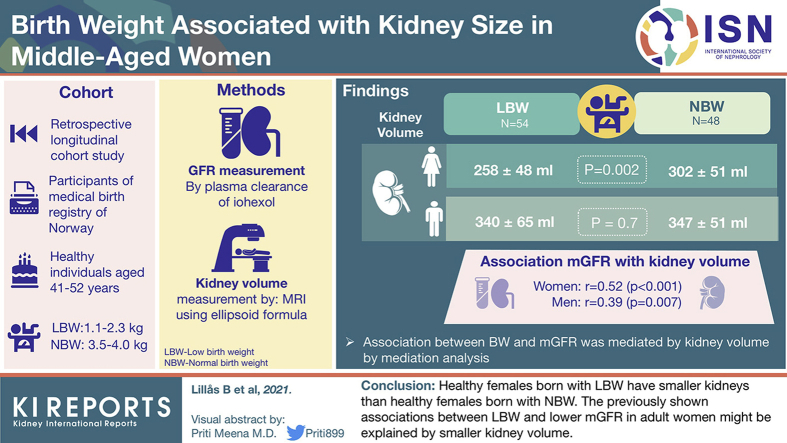

See Commentary on Page 2740

Over the years, an increasing amount of evidence has linked fetal life to risk of disease later in life.^1^, ^2^, ^3^, ^4^, ^5^ In nephrology, low birth weight (LBW), small for gestational age (SGA), and prematurity have been shown to associate with increased risk of albuminuria,^6^ hypertension,^7^ chronic kidney disease,^8^ and kidney failure.^9^ It is believed that low nephron number at birth is compensated by hyperfiltration of the remaining nephrons.^10^, ^11^, ^12^ The result is increased vulnerability of the nephrons for future insults.

Although the proposed mechanism involves a lower number of nephrons, this is difficult to measure *in vivo.* Kidney size has been used as a proxy,^11^ and indeed differences in kidney volume related to birth weight have been shown in children and young adults.^13^^,^^14^ However, the natural aging process and changes in kidney size with age may be different between men and women.^15^ In a previous study,^16^ we showed how measured GFR (mGFR) was different in healthy adult women with LBW versus normal birth weight (NBW). The same difference was not seen for men. One possible mechanism could be differences in kidney size.

In the present study, we aimed to examine the difference in kidney volume measured by magnetic resonance imaging (MRI) between LBW and NBW in healthy middle-aged adults, and to explore the relationship between kidney volume and mGFR. We further wanted to investigate the relationship between kidney volume and risk markers of cardiovascular and kidney disease, and to compare these findings with the effect of birth weight and birth weight for gestational age. To further add clinical value. we compared kidney size measurements using MRI with kidney size measurements on ultrasonography (US).

## Materials and Methods

### Study Design

This was part of a retrospective longitudinal cohort study comparing individuals with LBW (birth weight ≤2300 g) to individuals with NBW (birth weight 3500−4000 g).

### Registries

The Medical Birth Registry of Norway (MBRN) selected participants invited into the study. The MBRN contains complete data from 1967 to the present day on all births and pregnancies terminated in Norway after the 12th week of gestation.^17^ The registry includes data on pregnancy duration, birth weight, prenatal and perinatal complications, and parental background information. Our study included data on birth weight, gestational age, birth weight by gestational age, length at birth, and presence of preeclampsia.

### Participants

All participants were born between 1967 and 1976 and were currently residing in Haugesund and surrounding area, on the west coast of Norway. Using data from the MBRN, we identified and invited 200 persons with LBW and 200 persons with NBW. A total of 105 individuals were included in the study. A complete description of the inclusion process is given in a previous paper.^16^ Three participants did not participate in the MRI study, 2 because of personal choice and 1 because of metal implants.

### Study Overview

Participation required attendance on 2 separate days. On the first day, the participants met while fasting in the morning. This day included fasting blood samples, measurement of glomerular filtration rate (GFR) using iohexol clearance, blood pressure measurement, US of the kidneys, and a questionnaire. The second day the study was conducted in the afternoon (at least 1 week later than the first day) and included MRI scanning of the kidneys.

### Exposure Variables

Birth weight, birth weight for gestational age (sex-stratified *z* score, given as units of SD from the mean), and gestational age were obtained from the MBRN. We defined premature birth as delivery before the 37th week of gestation. Educational level, smoking status, and exercise frequency were self-reported in a questionnaire.

Height was measured and rounded to the nearest centimeter. Weight and body composition were measured using Tanita Body Composition Analyzer BC-418 (Tanita Corporation, Tokyo, Japan). Body mass index (BMI), body surface area (BSA; according to the formula by Du Bois), fat-free mass, and fat percentage were calculated directly by the machine.

The GFR was measured using plasma clearance of iohexol with blood samples at 2 and 4 hours, according to the method described by Jødal and Brøchner-Mortenssen.^18^ Blood pressure was measured 3 times during the first 30 minutes with the subject in a seated position, following the injection of iohexol, and the mean of the 2 latter measurements was used for analysis.

### Outcome Variables

Magnetic resonance imaging was performed using a 1.5 T scanner (Siemens Aera, Erlangen, Germany). Participants used oxygen during the examination and were instructed to breathe as shallowly as possible to avoid movement artifacts. This was part of a larger protocol and included examinations with and without contrast. The protocol consisted of axial T1 in and out phase, T2, diffusion imaging and coronal T1 with fat saturation and different flip angles, dynamic contrast-enhanced sequence (DCE-MRI), and delayed T1 fat-saturated sequence.^19^ Kidney size measurements were obtained on the most representative images of the basic axial T2 images and coronal T1 images with fat saturation without contrast enhancement. Kidney length, as well as 2 parenchymal thickness measurements, were measured on the coronal images, whereas width and depth (90° to each other) were measured on the axial images. Parenchymal thickness was measured as the distance from the outer renal capsule to the outermost start of the renal pelvis. Supplementary Figure S1A and B show examples of the measurements.

Kidney volume from the magnetic resonance images was calculated using the formula for simple ellipsoid equation: π/6 × length × width × depth. The volume of the renal pelvis was calculated as an ellipsoid with length, width, and depth calculated by subtracting 2 ∗ mean parenchymal width from the total kidney length, width, and depth (e.g., pelvic length = total kidney length – 2 ∗ mean parenchymal width). Parenchymal volume was calculated by subtracting the renal pelvis from the whole kidney. Total kidney volume (TKV) was the volume of the 2 kidneys combined.

Ultrasound was performed using Sonosite X2 apparatus (Fujifilm Sonosite Inc., Bothell, WA) with a C60xi, 5-2 MHz, curved array abdominal probe. We used a dorsal approach with the participant lying in the prone position. All measurements were taken on the maximal sonographic longitudinal view. We measured length and width, as well as 2 measurements of parenchymal width (Supplementary Figure S1C). In the same image, we manually traced the area of the whole kidney and the area of the renal pelvis.

Parenchymal area was calculated from the US images by subtracting the area of the pelvis from the area of the whole kidney.

### Statistical Analysis

All statistical analyses were performed using R version 4.0.2 (R Foundation for Statistical Computing, Vienna, Austria).^20^ Characteristics of included participants were compared between the LBW and NBW groups, and most of the analyses were performed separately for men and women. Results are presented for kidney measurements performed on both MRI and US, but the main analyses stem from the MRI measurements. Normally distributed data are shown as mean ± SD, and non−normally distributed data as median (minimum, maximum). A significance level of 0.05 was chosen for all tests. We used the Student *t* test for continuous data, and χ^2^ for categorical data. A linear regression model was fitted using total kidney volume as dependent variable. In this model, all measurements of body size were highly associated with total kidney volume. We therefore normalized kidney volume to 1.73 m^2^ BSA, and this was used as the dependent variable in the final linear regression model. Correlations between MRI and US kidney measurements were estimated using the Pearson correlation coefficient *r* and in a Bland−Altman plot. A mediation analysis was conducted according to Baron and Kenny^21^ with subsequent nonparametric bootstrap to estimate the confidence interval of the indirect effect. Birth weight per 100 g was used as the independent variable, kidney volume normalized for 1.73 m^2^ as the mediator, and mGFR in ml/min per 1.73m^2^ as the dependent variable. This analysis was performed for both the total sample and the sex-stratified sample. We used the R package “mediation” (version 4.5.0) with the percentile method using 5000 simulations.

### Ethics

This study was approved by the regional ethical committee (REK2017/927), and all participants provided signed written consent before participation. The study was conducted according to the Declaration of Helsinki.

## Results

We included 102 participants in the MRI study, 46 male, and 56 female; 54 were born LBW and 48 NBW. In addition to the difference in birth weight, the LBW group had lower birth weight for gestational age (−1.2 vs. 0.2, *P* < 0.001) and lower gestational age (34.5 weeks vs. 40.3, *P* < 0.001) (details are given in Table 1). On examination, the LBW group was shorter (170 cm vs. 173 cm, *P* = 0.04) than the NBW group, with no other significant differences in body size. The group with LBW also had higher blood pressure and a nonsignificant trend toward a lower mGFR as compared with those in the NBW group. In an earlier paper, we showed that women with LBW had significant lower mGFR than women with NBW (90.4 ± 12.2 vs. 101.0 ± 14.0, *P* = 0.005). No difference was seen in men (101.0 ± 14.5 vs. 100.0 ± 11.2, *P =* 0.7).^16^

**Table 1:** Characteristics of participants at birth and examination

Characteristic	Male individuals		Female individuals		*P* (group)	*P* (sex)
	LBW	NBW	LBW	NBW		
No. of participants	22	24	32	24		
Birth weight, g	2010 (1410, 2300)	3730 (3530, 3950)	2000 (1160, 2250)	3740 (3520, 3980)	<0.001	0.3
Birth weight for gestational age, SD	–0.9 (–3.9, 1.8)	0.1 (–0.4, 1.4)	–1.2 (–4.7, 1.1)	0.4 (–0.3, 1.5)	<0.001	0.5
Premature^a^	16 (73%)	0 (0%)	22 (69%)	0 (0%)	<0.001	0.7
Gestational age, wk^a^	34.2 ± 3.26	40.2 ± 1.74	34.7 ± 3.52	40.3 ± 1.03	<0.001	0.8
Maternal preeclampsia	4 (18%)	1 (4%)	3 (9%)	0 (0%)	0.09	0.5
Birth length, cm	44 (39, 49)	52 (48, 54)	45 (37, 48)	51 (48, 54)	<0.001	0.3
Age, yr	48 (41, 51)	47 (44, 51)	48 (42, 52)	46 (41, 50)	0.4	0.6
Height, cm	177 ± 7.3	179 ± 5.4	164.8 ± 4.4	167.5 ± 5.8	0.04	<0.001
Weight, kg	84.3 ± 12	83.8 ± 10.1	71.5 ± 15.3	74.1 ± 15.6	0.4	<0.001
Body mass index	27 (21.7, 34.3)	25.2 (21.2, 33.7)	25.4 (17.9, 37.6)	25.6 (20.7, 41.3)	0.8	0.9
Body surface area	2.01 ± 0.16	2.02 ± 0.12	1.78 ± 0.17	1.83 ± 0.18	0.2	<0.001
Fat-free mass	64 ± 7	65 ± 6	45 ± 5	48 ± 5	0.09	<0.001
Fat percentage	23.7 ± 6.1	21.5 ± 5.2	35.2 ± 7.7	34.4 ± 8.1	0.2	<0.001
Systolic blood pressure, mm Hg	131 ± 19	120 ± 8	123 ± 16	118 ± 14	0.02	0.1
Diastolic blood pressure, mm Hg	81 ± 12	72 ± 8	73 ± 11	67 ± 9	0.004	0.006
Measured GFR	102 ± 14	100 ± 11	90 ± 13	101 ± 14	0.06	0.01
Median albumin creatinine ratio	0.4 ± 0.7	0.4 ± 0.5	0.3 ± 0.4	0.3 ± 0.3	0.7	0.3
Completed higher education	12 (55%)	17 (71%)	14 (44%)	12 (50%)	0.3	0.1
Regular smoker	7 (32%)	0 (0%)	5 (16%)	6 (25%)	0.3	0.7
Exercise at least once a week	10 (45%)	4 (17%)	5 (16%)	5 (21%)	0.4	0.2

Comparisons between LBW and NBW (group) and between Male and Female (sex) were tested using χ^2^ for categorical data and Student *t* test for continuous data. *P* values shown. Normally distributed data are written as mean ± SD, non-normally distributed data as median (minimum, maximum), and categorical data as n (%).

GFR, glomerular filtration rate. LBW, low birth weight; NBW, normal birth weight.

a One female NBW individual had missing gestational age.

**Figure 1:**
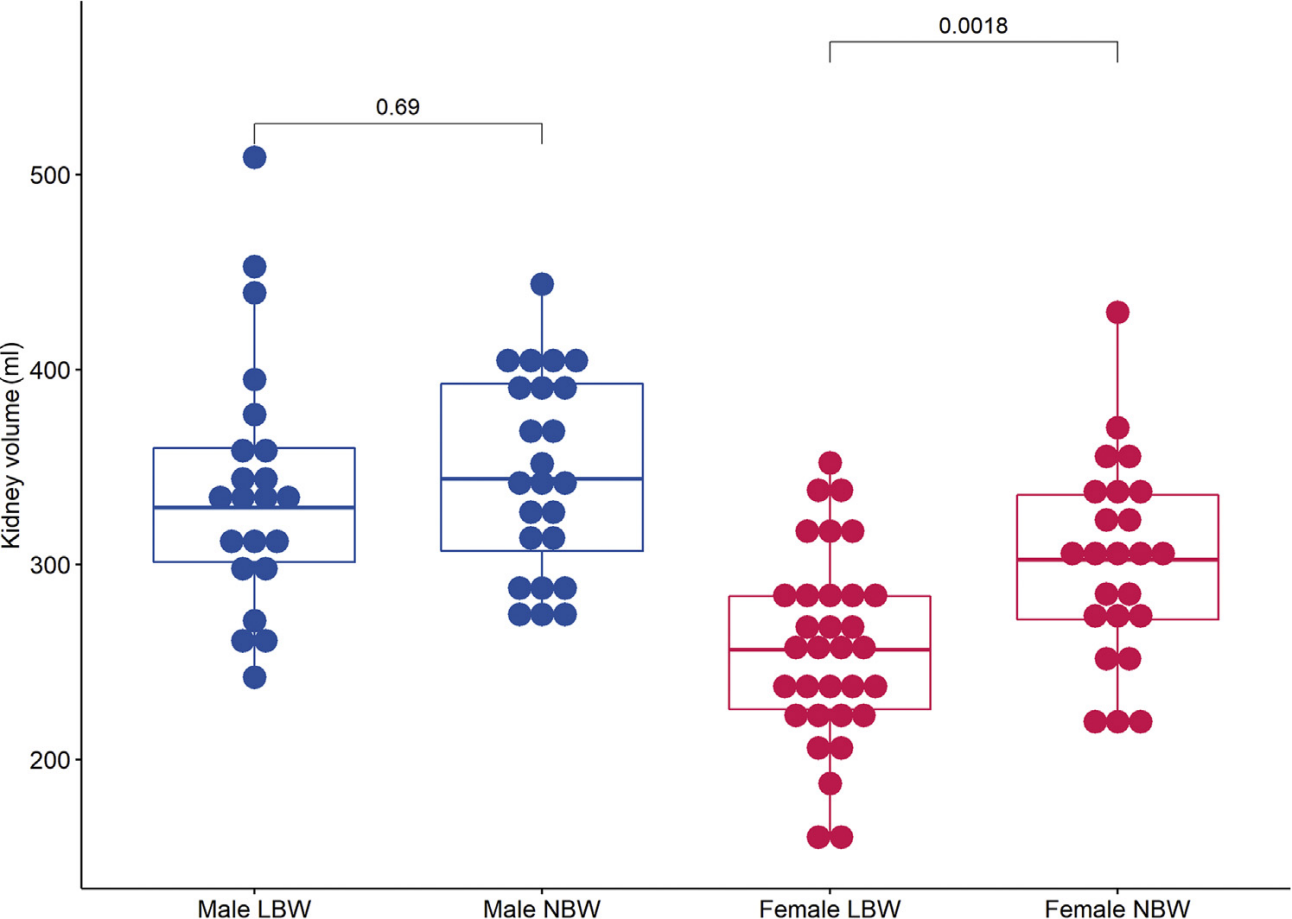
Distribution of kidney volume comparing low birth weight (LBW) and normal birth weight (NBW). Boxplot showing distribution of kidney volume stratified for sex and birth weight group. Sex-stratified comparison between birth weight groups was done using the Student *t* test, and *P* value is shown.

In the overall analyses, men had larger kidneys than women. This included measurements of kidney length, TKV, and kidney volume adjusted for BSA (*P* < 0.001 for all comparisons; Supplementary Table S1 provides details). When comparing the birth weight groups, there was a sex difference, with no difference in kidney size between the birth weight groups for the male participants, whereas for the female participants individuals with LBW had smaller kidneys than those with NBW. As shown in Figure 1 and Supplementary Table S1, the difference in kidney volume for women was significant for TKV and kidney volume per 1.73 m^2^ BSA.

**Figure 2:**
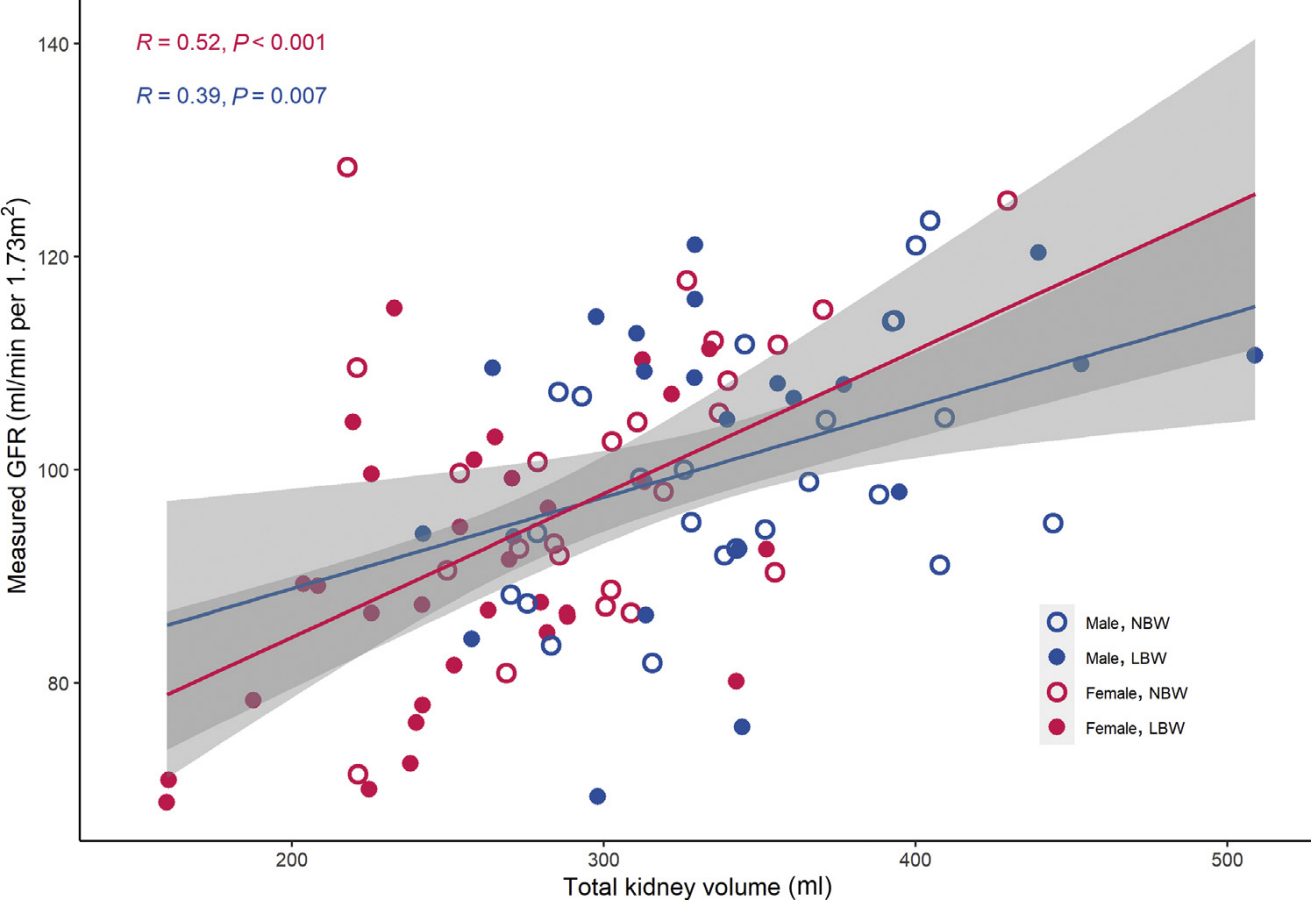
Relationship between kidney volume and measured glomerular filtration rate (GFR). Sex-stratified regression lines between kidney volume and measured GFR shown in pink (female) and blue (male). Sex-stratified correlation coefficient (Pearson *r*) and *P* value are shown.

In a linear regression model with TKV as the dependent variable, we found that TKV was highly dependent on the analyzed body size variables (height, weight, BMI, BSA, and fat-free mass). This was statistically significant both in the total sample and in sex-stratified analyses (*P* < 0.001 for all associations, except BMI in women [*P* = 0.001] and height in men [*P* = 0.8]). To avoid these confounding effects, we used kidney volume normalized for 1.73 m^2^ BSA as the dependent variable in the final model.^22^ This model shows that mGFR is associated with kidney volume, both for the total sample and for the sex-stratified groups (Table 2 and Figure 2). Being born LBW and birth weight for gestational age were both significantly associated with TKV in the total sample (*P* = 0.006 and *P =* 0.001, respectively) and in the female group (both *P* = 0.001), whereas in the male group this was not seen (*P* = 0.7 and 0.2, respectively). Being born preterm was not significantly associated with kidney volume; however, in the female subgroup, a nonsignificant trend toward smaller kidneys for those born preterm was seen (*P* = 0.08). Traditional kidney-related variables such as blood pressure and albuminuria showed no significant association with kidney volume, and this was also true for socioeconomic risk factors, smoking, and exercise (not shown).

**Table 2:** Sex-stratified linear regression model of kidney volume per 1.73 m^2^ as measured by magnetic resonance imaging

Characteristic	Male individuals			Female individuals		
	Estimate	*P* value	*R*	Estimate	*P* value	*R*
Birth weight, per increase of 100 g	0.38	0.6	0.08	1.95	<0.001	0.45
Birth weight by gestational age, per increase of 1 SD	6.13	0.2	0.18	12.37	0.001	0.42
Gestational age, per week of gestation	0.05	1	0	2.21	0.1	0.21
Body mass index, per increase of 1 kg/m^2^	4.9	0.01	0.38	–0.2	0.9	–0.02
Systolic blood pressure, per increase of 10 mm Hg	–2.3	0.6	–0.08	–0.4	0.9	–0.02
Diastolic blood pressure, per increase of 10 mm Hg	–2.2	0.7	–0.06	–1.2	0.8	–0.03
Measured GFR, per increase of 1 ml/min per 1.73 m^2^	1.5	0.003	0.43	1.6	<0.001	0.54
Median albumin creatinine ratio, per increase of 1 mg/mmol	10.6	0.4	0.14	11.7	0.5	0.1

GFR, glomerular filtration rate.

**Figure 3:**
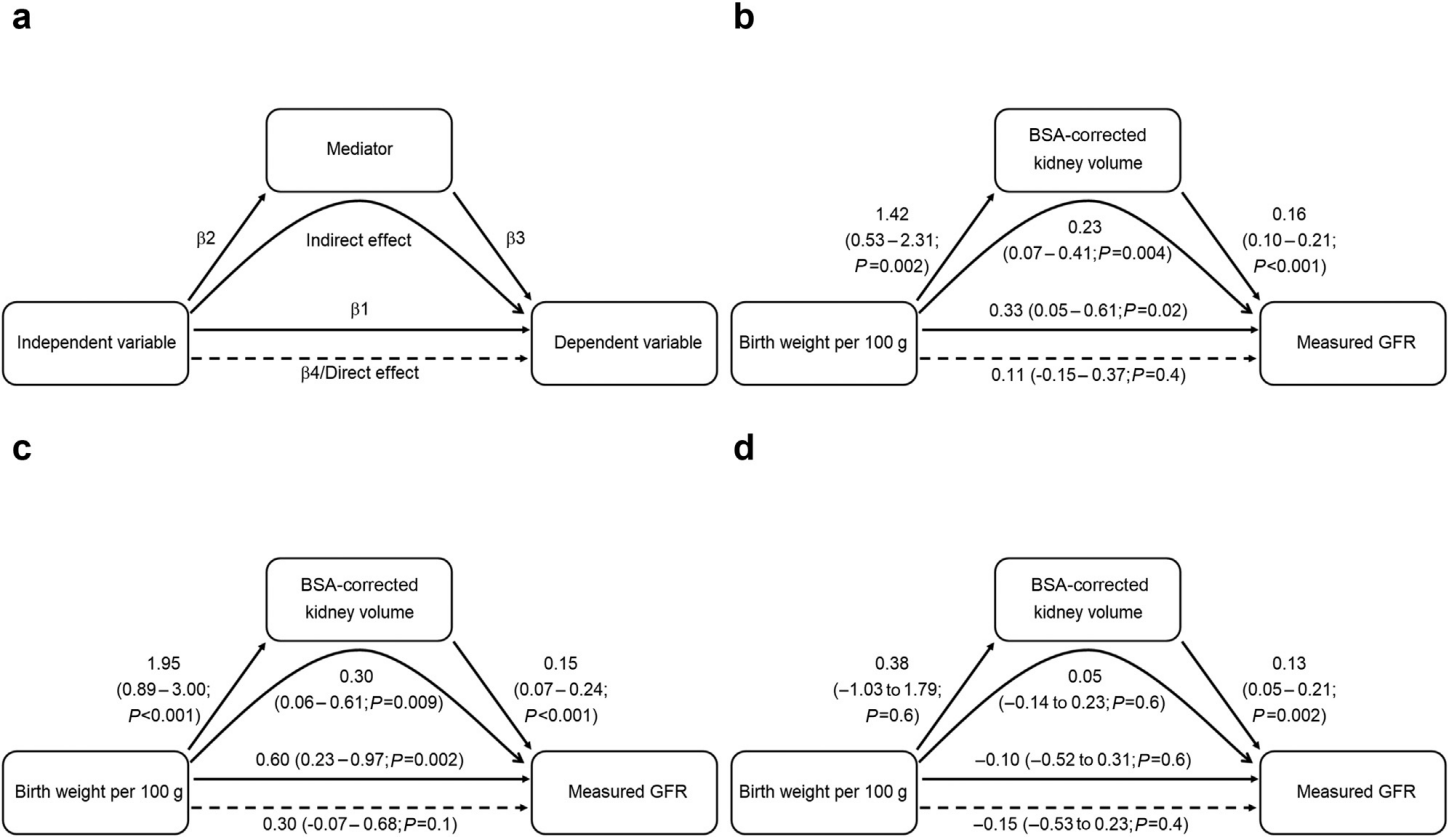
Kidney volume mediates effect of birth weight on measured glomerular filtration rate (GFR). (a) Example model, (b) total sample, (c) female individuals only, and (d) male individuals only. Each figure represents 3 regression equations: (i) independent variable → dependent variable, β1 is the effect estimate (with 95% confidence interval); (ii) independent variable → mediator, with β2 as the effect estimate; and iii) independent variable and mediator → dependent variable, with β3 as the effect estimate of the mediator and β4 as the effect estimate of the independent variable. The direct effect of the independent variable is the same as β4, whereas the indirect (mediated) effect, is β2 ∗ β3 or also β4 – β1. As shown in the figure, the effect of birth weight on measured GFR (significant β1 in women and total sample) was in fact mediated by the kidney volume (indirect effect).

To investigate whether the association between birth weight and mGFR was mediated by kidney volume, we performed a mediation analysis. The concept of a mediation analysis is outlined in Figure 3a. In the total sample, there was a significant association between birth weight and mGFR, as well as between birth weight and kidney volume (Figure 3b). When including both birth weight and kidney volume in the prediction model of mGFR, the effect estimate of birth weight was reduced, whereas the effect of kidney volume was significant. This suggests that a mediation occurred. The indirect (mediated) effect was calculated to 0.23, with the 95% confidence interval estimated after bootstrapping with 5000 simulations to 0.07 to 0.41 (*P* = 0.004). In women, a similar but somewhat stronger effect was seen (Figure 3c). In men, there was no significant association between birth weight and kidney volume and therefore no basis to perform a mediation analysis. This was, however, included in the figure for completeness (Figure 3d). Interestingly, the association between kidney volume and mGFR in men was similar to that in women, even when adjusted for birth weight.

A total of 82 participants had US measurements of acceptable quality to be included in analysis. The US parenchymal area was highly correlated with kidney volume as measured by MRI (*R* = 0.78, *P* < 0.001; see Supplementary Figure S2). Kidney length measurements were somewhat longer on MRI measurements than on US (4.5 ± 6.1 mm). This was probably due to underestimation on US length because of difficulties obtaining plane with maximum kidney length. The US measurements showed significant differences between sexes for both area and kidney length (*P* < 0.001), whereas kidney length in women was the only variable showing differences between the birth weight groups (*P* = 0.05) (see Supplementary Table S1).

## Discussion

We have previously shown that middle-aged women born with LBW had lower mGFR than middle-aged women born with NBW. The present paper uses the same cohort but analyzes the magnetic resonance images that were obtained as part of the study. We show that middle-aged women born with LBW also have smaller kidneys than middle-aged women born with NBW. We found that both birth weight and birth weight for gestational age were significantly associated with kidney size, whereas gestational age was not. No association between kidney size and birth weight was found in men with the same age and other similar characteristics. A mediation analysis showed that the effect of LBW on mGFR in the total sample and in women was mediated through kidney volume. We further used US for comparison with the MRI measurements, but even though the findings were similar, the differences in kidney size did not reach statistical significance between LBW and NBW individuals.

In our study, kidney volume was larger in men than in women, as has also been shown in previous studies.^23^^,^^24^ This effect was partly related to differences in BSA. Kidney size is closely related to body size,^23^, ^24^, ^25^ and to accommodate for this we chose to normalize kidney volume to BSA. In larger population-based studies, kidney size is shown to decrease with age.^15^^,^^25^^,^^26^ Kidney volume has been shown to correlate with birth weight.^27^ Premature newborns and those born small for gestational age have smaller kidneys than do term infants born appropriate for gestational age at birth.^28^^,^^29^ In very premature infants, there is some catch-up in kidney growth after birth; however, kidney size does not normalize.^28^ Smaller kidneys have been shown in preschool- and school-aged children born with LBW,^22^^,^^30^ although 1 Swedish study reports that this difference was lost after adjusting kidney volume for BSA.^31^ Extremely LBW individuals have smaller kidneys than very LBW individuals, suggesting a dose−response relationship.^32^^,^^33^ In a Dutch study of young adults, the difference in kidney size was still present; however, the authors found that this relationship was weaker in men than in women.^14^ Our findings show that the difference in kidney size related to birth weight, and possibly the difference in effect between the sexes, persist into at least the fifth decade. In women born LBW, birth weight per 100 g and birth weight for gestational age were associated with TKV, whereas prematurity showed only a nonsignificant trend. In men, no birth-related variable was associated with kidney volume. We are unsure of the underlying explanation for the sex difference; however, this may be the result of mechanisms both *in utero* and in childhood and early adult life. An autopsy study of non−growth-restricted fetuses showed that glomerular size in females was associated with gestational age, birth weight, kidney weight, and number of glomerular generations (indicator of total glomerular number), whereas this was not seen in males.^34^ A possible explanation of our findings may be that kidney growth is more dependent on birth-related variables in women than in men. However, a previous study in rats found that female offspring were more resistant to protein restriction during pregnancy than male offspring.^35^ Similarly, differences in the natural age-related changes in kidney size between males and females may be relevant. Studies have shown that age-related structural changes of the kidneys differs somewhat between men and women.^36^^,^^37^ In women, kidney size seems to be stable up to about the fourth decade before a decline starts, whereas in men there seems to be an increase to about the fifth decade before a decline starts.^15^^,^^25^

Kidney volume is sometimes used as a proxy for nephron number, and kidney weight is shown to correspond to nephron number.^38^ The more precise method of measuring nephron number by stereology can be done only at autopsy,^39^^,^^40^ and kidney biopsies combined with contrast-enhanced imaging^36^^,^^41^ are too invasive for healthy volunteers. A promising method using cationized ferritin in MRI is still only experimental.^42^^,^^43^ The validity of the approximation between kidney size and nephron number has, however, been questioned.^44^ As a result, the exact meaning of kidney size is uncertain. In adult polycystic kidney disease, height-adjusted total kidney volume is a prognostic factor,^45^ and in kidney transplantation, size certainly matters.^46^^,^^47^ Previous studies have shown kidney size to be correlated with kidney function.^23^^,^^24^^,^^48^^,^^49^ We also found this and observed the same increase in kidney volume per increase in GFR of 1 ml/min per 1.73 m^2^ for men (β = 1.5) and women (β = 1.6), although there was a higher correlation coefficient in women than in men (*r* = 0.54 vs. *r* = 0.43). Glomerular filtration rate is a functionally more important variable than kidney volume, and there is a need for a better understanding of the underlying mechanisms between birth weight, kidney volume, and kidney function. In the present study, we performed a mediation analysis and showed that the association between birth weight and kidney function was in fact mediated by kidney volume. Given the hypothesis that kidney volume could reflect nephron number, this is a possible pathophysiological mechanism for the association between birth weight and GFR. We did not find any association between kidney volume and traditional kidney risk factors such as blood pressure or albuminuria in our healthy population sample. This in contrast to the Framingham Heart Study, in which both hypertension and albuminuria were found to be associated with larger TKV.^23^ In 2 other studies, hypertension was not associated with kidney volume.^50^^,^^51^

Imaging of the kidneys can be done using various methods, such as MRI, US, and computed tomography (CT), and there is no clear answer as to which is the best method. We chose MRI as part of a larger protocol, with readily accessible measurements of kidney size. The ellipsoid method is an easily available method for estimating kidney volume. It is prone to error, and usually underestimates the true kidney volume.^52^ However, when using the same method for the whole sample, and especially with the investigator blinded to BW group, we believe that this does not affect the outcome. To further add clinical value we used bedside ultrasonography to measure kidney size. We chose a dorsal approach, with the participants lying in the prone position, believing that the dorsal measurements give a more standardized view of the left and right kidney compared to measurements performed in the lateral view. Similar findings in healthy medical students were made by our colleagues, with lower intra- and interobserver variation with the dorsal measurements (Eikrem *et al.*, unpublished data). The method showed good correlation with MRI kidney volume based on the ellipsoid formula (*r* = 0.78, *P* < 0.001). When comparing the kidney length on the magnetic resonance and US images, we found shorter length on the US. We believe that this is due to difficulty in finding the maximum kidney length using US. The measurements with US did not, however, show the same statistical significance for the birth weight groups as did MRI, most likely due to lower precision of measurements and difficulties obtaining the plane of maximum length. From our results, it appears that differences in kidney size between LBW and NBW groups should be examined by MRI or CT scanning, and that US might be too imprecise.

A strength of the present study is that we used a national birth registry to obtain high-quality data on birth-related variables. The fact that we used measured rather than estimated GFR is another strength, as this is a more accurate measurement, especially for participants with values in the normal range. We studied a group with birth weight <2.3 kg and compared this with a group with high normal birth weight (3.5−4.0 kg). This allowed for higher statistical power in the comparison between low and normal birth weight; however, a weakness of this approach is that we cannot investigate the continuum between these groups. Other weaknesses include using the ellipsoid method for volume estimation, which, although common in use, is prone to error. This method usually underestimates the true kidney volume^52^; however, we believe that by using the same method for all participants, the consistent underestimation of the ellipsoid method should not affect the results. A less experienced operator performed the ultrasound measurements; nevertheless, only those measurements with satisfying quality as assessed by another operator were included in the study.

We conclude that kidney volume is significantly associated with birth weight in middle-aged women, but not in men. This difference in kidney volume may indeed explain the observed sex difference in the association between birth weight and measured GFR. More studies differentiating cortex and medulla, as well as studies including kidney histology, are needed. Studies investigating changes in kidney size through adulthood also seem warranted, to address the sex difference in our study.

## Disclosure

All the authors declared no competing interests.

## Supplementary Material

Supplementary File (PDF)

**Table S1.** MRI and US kidney measurements by sex and birth weight group

**Figure S1.** Kidney measurements using MRI and US.

**Figure S2.** Correlation between MRI and US measurements.
