# Supplementary material for: Birth Weight Is Associated With Kidney Size in Middle-Aged Women
Source: Kidney Int Rep. 2021 Sep 7;6(11):2794–802. doi: 10.1016/j.ekir.2021.08.029 (PMC8589725; doi:10.1016/j.ekir.2021.08.029)
Supplement: Supplementary File (PDF) [file mmc1.pdf]

Supplementary materials

Supplementary table S1 - MRI and US kidney measurements by sex and birth weight group

	Total			Male			Female		
				LBW	NBW		LBW	NBW	
	Mean ± SD	p (group)	p (sex)	Mean ± SD	Mean ± SD	p-value	Mean ± SD	Mean ± SD	p-value
Measurements from MRI									
Number of participants - n	102			22	24		32	24	
Volume left kidney – mL	155.2 ± 35.6	0.1	<0.001	178.3 ± 34.2	174.3 ± 26.8	0.7	130.6 ± 28.6	147.8 ± 29.9	0.04
Volume right kidney – mL	151.5 ± 32.6	<0.001	<0.001	161.4 ± 35.4	172.5 ± 26.9	0.2	127 ± 22.6	154.2 ± 26.6	<0.001
Total kidney volume – mL	306.7 ± 64.5	0.008	<0.001	339.7 ± 65.2	346.8 ± 50.9	0.7	257.6 ± 48.1	302 ± 50.9	0.002
Mean kidney length – cm	11.1 ± 0.8	0.1	<0.001	11.5 ± 0.6	11.6 ± 0.6	0.8	10.6 ± 0.9	10.9 ± 0.8	0.2
Mean parenchymal width – cm	1.8 ± 0.2	0.5	<0.001	1.9 ± 0.2	1.8 ± 0.2	0.2	1.7 ± 0.2	1.7 ± 0.2	0.1
Kidney volume per 1.73m^2 - mL/1.73m^2	278.4 ± 44.0	0.005	<0.001	291.4 ± 45.2	296.4 ± 41.1	0.7	250.5 ± 38.6	285.7 ± 36.0	<0.001
Measurements from US									
Number of participants – n	82			18	19		25	20	
Area of both kidneys combined - cm^2	62.6 ± 10.0	0.4	<0.001	69.4 ± 9.5	67.6 ± 8.2	0.5	56.2 ± 8.5	59.8 ± 7.6	0.1
Mean kidney length – cm	10.6 ± 0.9	0.2	<0.001	11.2 ± 0.8	11.1 ± 0.6	0.6	10.0 ± 0.8	10.4 ± 0.6	0.05
Mean parenchymal width – cm	1.4 ± 0.2	0.8	<0.001	1.5 ± 0.2	1.5 ± 0.1	0.4	1.3 ± 0.1	1.3 ± 0.1	0.9

Measurements from MRI were done for all included participants. Measurements from US only for those with acceptable quality of images. Comparison using student's t-test was performed. In the total sample comparing both birth weight group and sex. In the sex-stratified comparing birth weight groups.

Supplementary figure S1 - Kidney measurements using MRI and US

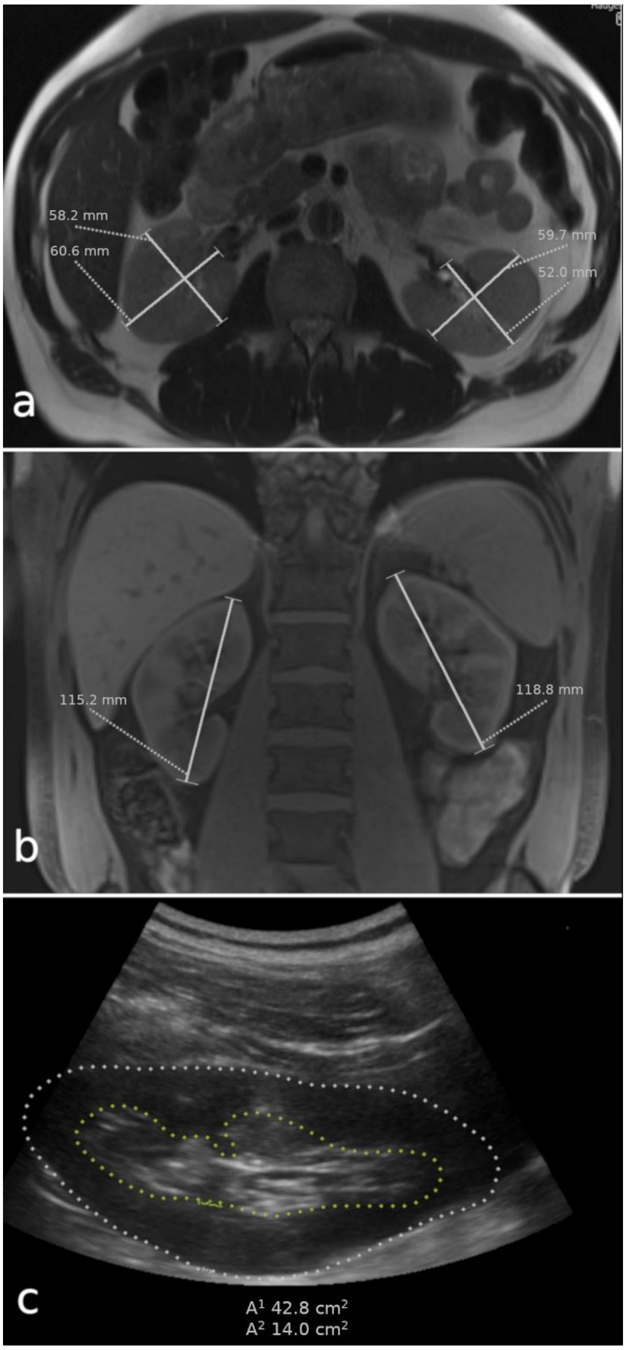

Legend:

Kidney volume was estimated using ellipsoid formula using measurements from MRI. (a) Basic axial T2 image with measurements of kidney width and depth. (b) coronal T1 image with measurement of kidney length. (c) Dorsal US image with hand-traced area of total kidney (A^1) and pelvis (A^2).

Supplementary figure S2 - Correlation between MRI and US measurements

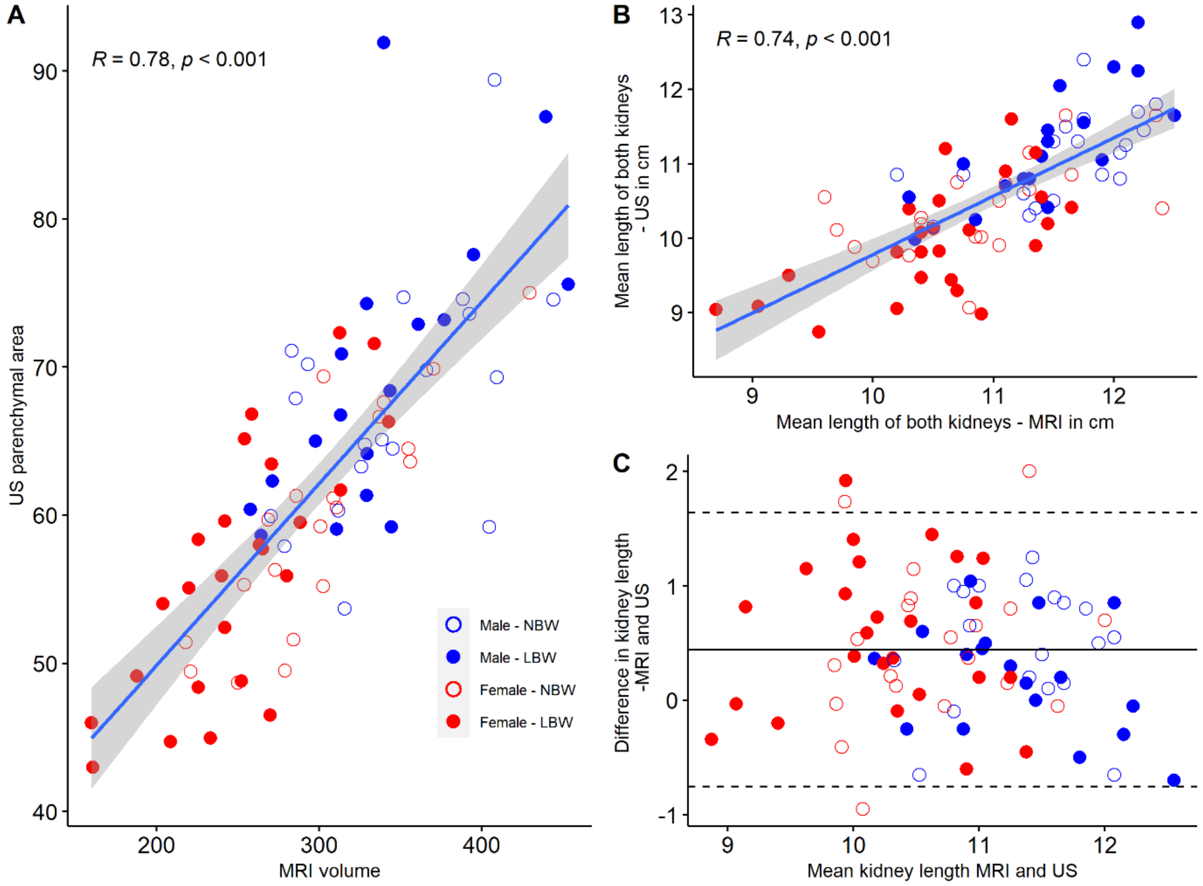

Legend

Kidney volume estimated by ellipsoid formula from MRI images correlated well with dorsal US parenchymal are. (a) Correlation between MRI volume and US parenchymal area, (b) correlation between mean kidney length measured by MRI and US, and (c) Bland Altman plot of mean kidney length measured by MRI and US.
